# Data about marine area-based management tools to assess their contribution to the UN sustainable development goals

**DOI:** 10.1016/j.dib.2021.107704

**Published:** 2021-12-11

**Authors:** Elena Gissi, Frank Maes, Zacharoula Kyriazi, Ana Ruiz-Frau, Catarina Frazão Santos, Barbara Neumann, Adriano Quintela, Fátima L. Alves, Simone Borg, Wenting Chen, Maria da Luz Fernandes, Maria Hadjimichael, Elisabetta Manea, Márcia Marques, Froukje Maria Platjouw, Michelle E. Portman, Lisa P. Sousa, Luca Bolognini, Wesley Flannery, Fabio Grati, Cristina Pita, Natașa Văidianu, Robert Stojanov, Jan van Tatenhove, Fiorenza Micheli, Anna-Katharina Hornidge, Sebastian Unger

**Affiliations:** a Hopkins Marine Station, Stanford University, 120 Ocean View Blvd, Pacific Grove, CA 93950, USA; b National Research Council, Institute of Marine Science, CNR ISMAR, Arsenale, Tesa 104 - Castello 2737/F, 30122 Venice, Italy; c University IUAV of Venice, Santa Croce 191, 30135 Venezia, Italy; d Faculty of Law and Criminology, Maritime Institute, Ghent University, Universiteitstraat 6, 9000 Ghent, Belgium; e Interdisciplinary Centre of Marine and Environmental Research (CIIMAR), University of Porto, Terminal de Cruzeiros de Leixões. Av. General Norton de Matos s/n, 4450-208 Matosinhos Portugal; f Department of Marine Ecosystem Dynamics, IMEDEA (CSIC-UIB), Miquel Marqués, 21, 07190, Esporles, Spain; g Marine and Environmental Sciences Centre, Faculdade de Ciências, Universidade de Lisboa, Avenida Nossa Senhora do Cabo 939, 2750-374 Cascais, Portugal; h Environmental Economics Knowledge Center, Nova School of Business and Economics, New University of Lisbon, Rua da Holanda 1, 2775-405 Car cavelos, Portugal; i Institute for Advanced Sustainability Studies (IASS), Berliner Str. 130, D-14467 Potsdam, Germany; j CESAM-Centre for Environmental and Marine Studies, Department of Environment and Planning, University of Aveiro, Campus Universitàrio de Santiago, 3810-193 Aveiro, Portugal; k Department of Environmental and Resources Law, University of Malta, Msida, MSD 2080, Malta; l Norwegian Institute for Water Research, Gaustadalléen 21, NO-0349 Oslo, Norway; m Independent Researcher, Nicosia, Cyprus; n Technion – Israel Institute of Technology, Kiryiat HaTechnion, Haifa 32000 Israel; o National Research Council (CNR), Institute of Marine Biological Resources and Biotechnologies (IRBIM), Largo Fiera della Pesca 1, 60125 Ancona, Italy; p School of Natural and Built Environment, David Keir Building, Queen's University Belfast, Belfast, BT9 5AG, United Kingdom; q International Institute for Environment and Development (IIED), 235 High Holborn, Holborn, London WC1V 7DN, U.K; r Faculty of Natural Sciences and Agricultural Sciences, Ovidius University of Constanța, Aleea Universității 1, 900470, Constanța, Romania; s Interdisciplinary Center for Advanced Research on Territorial Dynamics, University of Bucharest, Regina Elisabeta 4-12, 030018, Bucharest, Romania; t Faculty of Business and Economis, Mendel University in Brno, Zemědělská 1, 61300 Brno, Czech Republic; u Centre for Blue Governance, Department of Planning, Aalborg University, Rendsburggade 14, 9000 Aalborg, Denmark; v Stanford Center for Ocean Solutions, 120 Ocean View Blvd, Pacific Grove, CA 93950, USA; w German Development Institute / Deutsches Institut für Entwicklungspolitik (DIE), Tulpenfeld 6, D - 53113 Bonn, Germany

**Keywords:** International and regional agreements, Marine protected areas, Shipping, Fisheries management, Marine spatial planning, Underwater cultural heritage, Deep seabed mining

## Abstract

The dataset presented in this article contains information about marine Area-Based Management Tools (ABMTs) used to assess their contribution to the United Nations 2030 Sustainable Development Goals. Following the scope of the analysis, ABMTs were identified by scrutinizing international and regional legal sources related to ocean management in the fields of marine conservation, fisheries, deep sea bed mining, underwater natural and cultural heritage, environmental conservation, and marine spatial planning. Legal sources were screened to depict the following characteristics of individual ABMTs: i) management objectives; ii) authorities responsible for delivering such objectives; iii) the system of management and planning entailed in the ABMT including the zoning type; and iv) the specific spatial scope and domain each ABMT refer to in vertical depth and horizontal domain. Data were generated through an internal expert elicitation. Experts, initially trained in the data analysis and related protocol, contributed to the data production because of their specific knowledge and experience in ocean management. This dataset represents a unique source of information for advancing research about monitoring and assessment of the achievement of sustainable development goals that encompasses different types of ABMTs.

## Specifications Table

**Table:** 

Subject	Environmental sciences → Management, Monitoring, Policy and Law
Specific subject area	Data pertain to the legal sources in force to the management and planning of the ocean and coastal areas in area under and beyond national jurisdiction.
Type of data	Table
How the data were acquired	An initial list of 47 ocean-related international agreements (at global and regional levels) was compiled, with respect to shipping, fisheries management, deep seabed mining in the Area, underwater natural and cultural heritage, environmental conservation, and marine spatial planning. We screened them and compiled a list of ABMTs mentioned by the respective legal sources and related tools. We recorded how legal sources at the international level have shaped ABMTs with regard to spatial scope, mandate and responsibilities, and single/multiple sector-based objectives. Data about ABMTs were collected with respect to: i) their objectives; ii) authorities responsible for delivering such objectives; iii) the system of management and planning entailed in the ABMT types; and iv) the specific spatial scope and domain each ABMT refer to in vertical depth and horizontal domain.
Data format	Analysed (the repositories where primary sources are hosted are all reported in the last column of the table in supplementary information)
Description of data collection	Of the initial full list of ocean-related international agreements, we retained only those responding to the following two criteria: i) implementation in practice; and ii) existing specific, identifiable geographical scope for zoning.
Data source location	Data source locations are reported in the Dataset table, column “Data source location, additional information, notes or web links”
Data accessibility	In this article
Related research article	*If accepted, the article to be cited as in press is the following:* E. Gissi, F. Maes, Z. Kyriazi, A. Ruiz-Frau, C. Frazão Santos, B. Neumann, A. Quintela, F. L. Alves, S. Borg, W. Chen, M. da Luz Fernandes, M. Hadjimichael, E. Manea, M. Marques, F. M. Platjouw, M. E. Portman, L. P. Sousa, L. Bolognini, W. Flannery, F. Grati, C. Pita, N. Văidianu, R. Stojanov, J. van Tatenhove, F. Micheli, A.K. Hornidge, S. Unger (2022) Contributions of marine area-based management tools to the UN Sustainable Development Goals, Journal of Cleaner Production, 330, 129910, DOI:10.1016/j.jclepro.2021.129910

## Value of the Data

• The value of this data derives from the systematic analysis of Area-based management tools (ABMT) proposed in this study providing a complete and unique source of information for further research, for instance, related to defining performance indicators for sustainable development goals to encompass the different types of ABMTs, whose characteristics are analysed and reported here.
• Decision makers, non-governmental organizations, practitioners and marine managers can use this dataset to understand, confront, and select the best area based management tool based on the characteristics resulting in this secondary dataset to address the specific management problem they need to address.
• This dataset can be used to set potential monitoring strategies and protocols based on the data collected here for the area-based management tools, for instance, to define a framework of indicators for the achievement of sustainable development goals.

## 1 Data Description

Data about Area-based management tools (ABMTs) and related legal sources from international and regional agreements. All the primary sources were retrieved from public institutional websites of the respective responsible authorities and accessed on March 9, 2020. The data protocol for the identification and description of ABMTs from legal documents and acts reports the following information: i) ABMT focus/sector or type of management defined under each tool, ii) reference to the legal source including short name of the source and/or name of the institution, iii) year of entry into force of the legal sources, iv) short definition as used in official documents (including references to article/s), v) instruments used to implement ABMT, vi) vertical marine subdivisions/zones to which ABMT applies, vii) brief outline of the maritime jurisdictional areas addressed, (according to UNCLOS [1]), viii) brief description or outline of sector/topic for establishing/ mentioning an ABMT, ix) Authority associated with the ABMT, x) list of tools and/or management strategies associated), xi) year of issuing and example(s) of implementation, xii) link to the primary legal source.

## 2 Experimental Design, Materials and Methods

Data were generated considering to identify ABMTs and related characteristics, then to assess their contribution to the United Nations Sustainable Development Goals [1]. We defined Area-Based Management Tools (ABMTs) as globally applied, purpose-orientated instruments used in the planning and management of marine and coastal areas, entailing the implementation of a system of rights and duties in a particular management area (spatially explicit), under the responsibility of a designated authority, to afford high levels of protection [2,3]. Since the system of rights and duties is enforced through legal sources, we search for ABMTs and data by selecting legal sources related to planning and management of marine and coastal areas for conservation, shipping, fisheries, deep seabed mining, and Underwater Cultural and Natural Heritage (UCNH) management, and Marine Spatial Planning (MSP).

Because of the global scope of the analysis, we identified legal sources at international (e.g., United Nations) and regional level (e.g., Regional Fishery Organizations), without addressing specific regulations at national level of individual States. Since of international and regional agreements covered all ocean, we consider the dataset representative of the ABMTs implemented globally.

The authors compiled an initial list of 58 ocean-related international agreements (at global and regional levels). We removed 11 international agreements that were not implemented yet, and obtained a final list of 47 international agreements.

We screened them and compiled a list of ABMTs mentioned by the respective legal sources and related tools. We recorded how legal sources at the international level have shaped ABMTs with regard to spatial scope, mandate and responsibilities, and single/multiple sector-based objectives. Data about ABMTs were collected with respect to: i) their management objectives; ii) authorities responsible for delivering such objectives; iii) the system of management and planning entailed in the ABMT forms; and iv) the specific spatial domain ABMTs refer to (both vertical depth and horizontal).

Data were generated through internal expert elicitation. Experts involved – as the authors of this study – were part of the Working Group on “Area Based Management” of the European COST Action CA 15217 OceanGov “Ocean Governance for Sustainability: Challenges, Options and the Role of Science”. The experts had a diverse and in-depth knowledge about the different Area-Based Management Tools (ABMTs) related to conservation, shipping, fisheries, deep seabed mining, Underwater Cultural and Natural Heritage (UCNH), and Marine Spatial Planning (MSP).

The experts, divided in groups by management sector, were asked to compile an initial list of international and regional legal sources for their management sector. The initial lists were then shared among the entire team to identify potential missing sources. Once the lists were defined, the data collection was performed in groups by annotating data from the legal sources on a shared spreadsheet. The groups were initially trained with a workshop held in Aveiro (Portugal) in June 2018. All the authors contribute to identifying the legal sources and revising the data collected remotely.

## Ethics Statements

The data collection and process does not involve any ethical concerns.

## CRediT Author Statement

**Elena Gissi** and **Frank Maes:** Conceived and structured the data collection, performed the first search of the legal sources of ABMTs; all the authors contributed to the data collection and data annotation.

## Declaration of Competing Interest

The authors declare that they have no known competing financial interests or personal relationships that could have appeared to influence the work reported in this paper.

## Appendix Supplementary Materials

Image, application 1
