# Supplementary material for: Data about marine area-based management tools to assess their contribution to the UN sustainable development goals
Source: Data Brief. 2021 Dec 11;40:107704. doi: 10.1016/j.dib.2021.107704 (PMC8685976; doi:10.1016/j.dib.2021.107704)
Supplement: Supplementary file 1 [file mmc1.docx]

**Data on marine area-based management tools to assess their contribution to the UN Sustainable Development Goals**

Elena Gissi^1,2,3^*, Frank Maes^4^, Zacharoula Kyriazi^5^, Ana Ruiz-Frau^6^, Catarina Frazão Santos^7,8^, Barbara Neumann^9^, Adriano Quintela^10^, Fátima L. Alves^10^, Simone Borg^11^, Wenting Chen^12^, Maria da Luz Fernandes^10^, Maria Hadjimichael^13^, Elisabetta Manea^2^, Márcia Marques^10^, Froukje Maria Platjouw^12^, Michelle E. Portman^14^, Lisa P. Sousa^10^, Luca Bolognini^15^, Wesley Flannery^16^, Fabio Grati^15^, Cristina Pita^10,17^, Natașa Văidianu^18,19^, Robert Stojanov^20^, Jan van Tatenhove^21^, Fiorenza Micheli^1,22^, Anna-Katharina Hornidge^23^, Sebastian Unger^9^

1 Hopkins Marine Station, Stanford University, Pacific Grove, CA 93950, USA

2 National Research Council, Institute of Marine Science, CNR ISMAR, Arsenale, Tesa 104 - Castello 2737/F, 30122 Venice – Italy

3 University IUAV of Venice, Santa Croce 191, 30135 Venezia, Italy

4 Faculty of Law and Criminology, Maritime Institute, Ghent University, Universiteitstraat 6, 9000 Ghent, Belgium

5 Interdisciplinary Centre of Marine and Environmental Research (CIIMAR), University of Porto, Terminal de Cruzeiros de Leixões. Av. General Norton de Matos s/n, 4450-208 Matosinhos Portugal

6 Department of Marine Ecosystem Dynamics, IMEDEA (CSIC-UIB), Miquel Marqués, 21, 07190, Esporles, Spain

7 Marine and Environmental Sciences Centre, Faculdade de Ciências, Universidade de Lisboa, Avenida Nossa Senhora do Cabo 939, 2750-374 Cascais, Portugal

8 Environmental Economics Knowledge Center, Nova School of Business and Economics, New University of Lisbon, Rua da Holanda 1, 2775-405 Car cavelos, Portugal

9 Institute for Advanced Sustainability Studies (IASS), Berliner Str. 130, D-14467 Potsdam, Germany

10 CESAM-Centre for Environmental and Marine Studies, Department of Environment and Planning, University of Aveiro, Campus Universitàrio de Santiago, 3810-193 Aveiro, Portugal

11 Department of Environmental and Resources Law, University of Malta, Msida, MSD 2080, Malta

12 Norwegian Institute for Water Research, Gaustadalléen 21, NO-0349 Oslo, Norway

13 Independent Researcher, Nicosia, Cyprus

14 Technion – Israel Institute of Technology, Kiryiat HaTechnion, Haifa 32000 Israel

15 National Research Council (CNR), Institute of Marine Biological Resources and Biotechnologies (IRBIM), Largo Fiera della Pesca 1, 60125 Ancona, Italy

16 School of Natural and Built Environment, David Keir Building, Queen’s University Belfast, Belfast, BT9 5AG, United Kingdom

17 International Institute for Environment and Development (IIED), 235 High Holborn, Holborn, London WC1V 7DN, U.K.

18 Faculty of Natural Sciences and Agricultural Sciences, Ovidius University of Constanța, Aleea Universității 1, 900470, Constanța, Romania

19 Interdisciplinary Center for Advanced Research on Territorial Dynamics, University of Bucharest, Regina Elisabeta 4-12, 030018, Bucharest, Romania

20 Faculty of Business and Economis, Mendel University in Brno, Zemědělská 1, 61300 Brno, Czech Republic

21 Centre for Blue Governance, Department of Planning, Aalborg University, Rendsburggade 14, 9000 Aalborg, Denmark

22 Stanford Center for Ocean Solutions, 120 Ocean View Blvd, Pacific Grove, CA 93950, USA

23 German Development Institute / Deutsches Institut für Entwicklungspolitik (DIE), Tulpenfeld 6, D - 53113 Bonn, Germany

* Corresponding author: Elena Gissi, Hopkins Marine Station, Stanford University, Pacific Grove, CA 93950, USA, tel: +1 831 655 6200; e-mail address: elena.gissi@ismar.cnr.it

Dataset: Review of Area-based management tools (ABMTs) and related legal sources from International and Regional Agreements. All the web links of Tab. 7 were accessed on March 9, 2020

| **No.** | **Code** | **Level** | **Legal source** | **Year of entry into force** | **Definition of Area Based Management (including references to article(s)** | **Instruments** | **Marine domain** | **Maritime jurisdictional area** | **Any formal identification of the sector/topics as the purpose of ABM** | **Is there any authority mentioned to establish/claim the ABMT?** | **Any tools mentioned?** | **Examples of implementation and year the tool was introduced** | **Data source location, additional information, notes or web links** |
| --- | --- | --- | --- | --- | --- | --- | --- | --- | --- | --- | --- | --- | --- |
| 1 | I | In | ISA (Areas of Particular Environmental Interest; also development of environmental management plans for defined areas such as the Clarion Clipperton Zone) | 2012 - The Environmental Management Plan (EMP). | "Area" means the seabed and ocean floor and subsoil thereof, beyond the limits of national jurisdiction; | [Rationale and recommendations for the establishment of preservation reference areas for nodule mining in the Clarion-Clipperton Zone (ISA Guidelines: ISBA/4/C/4/Rev. 1, annex 4, section 5.6.)](https://www.isa.org.jm/files/documents/EN/14Sess/LTC/ISBA-14LTC-2.pdf) | Subsoilseabedocean floor | Mineral resources in "The Area" | References Areas for Deep Seabed Mining; part of a regional Environmental Management Plan to be developed and implemented by the ISA | ISA (Mining Code currently under development in ISA) | 21. The conditions of paragraph 8 will expire 30 years from entry into force of this measure, unless the Commission decides to reaffirm or modify the conditions of paragraph 8 based on the Scientific Committee advice provided under paragraph 17. If the provisions of paragraph 8 expire, unless otherwise decided, the catch limit in the area defined by the boundaries of the Special Research Zone shall not exceed 20% of the total catch limit for Statistical Subarea 88.1 and SSRUs 882 A–B, combined. | Clarion Clipperteron Zone (South Pacific) | https://www.isa.org.jm/files/documents/EN/14Sess/LTC/ISBA-14LTC-2.pdf |
| 2 | S | In | UNCLOS art. 211.6 | 1994 | Clearly defined area: Although called "clearly defined area" and also "special area" in UNCLOS, these areas are not limited to special areas under MARPOL. In fact, these special areas under art. 211.6 are more related to PSSAs (particular sensitive sea areas) if in clearly defined areas in the EEZ of coastal states or partly beyond the territorial sea. The "clearly defined areas" are however more limited in scope than PSSAs: prevention of pollution from vessels. | limits of 'clearly defined area' (not clear if charts or special areas are meant) | Water columnsurface | National jurisdiction in EEZ: balance between sovereign rights coastal states (EEZ) + flag states within IMO (freedom of navigation). | Clearly defined areas' aim to prevent, reduce and control pollution from vessels | The coastal state(s), the IMO, and flag states together adopt the area and special mandatory measures to prevent pollution from vessels due to oceanographic and ecological conditions of that area, as well as for the protection of the resources of the coastal state. | Charts and geographical coordinates: discharge limitations and navigational practices for foreign vessels | There are 17 PSSAs, located all over the world. Not all apply in the EEZ. The ones that (partly) do are: The Great Barrier Reef, Australia (designated a PSSA in 1990); The Sabana-Camagüey Archipelago in Cuba (1997); Malpelo Island, Colombia (2002); Western European Waters (2004); The Baltic Sea area, Denmark, Estonia, Finland, Germany, Latvia, Lithuania, Poland and Sweden (2005); The Papahānaumokuākea Marine National Monument, United States (2007); Extension of Great Barrier Reef and Torres Strait to encompass the south-west part of the Coral Sea (2015); The Jomard Entrance, Papua New Guinea (2016) | www.pssa.imo.org |
| 3 | S | In | IMO (PSSAs) (see also supra UNCLOS art. 211.6)  *(IMO Resolution A. 720 (17), 06.11.1991), although the Great Barrier Reef was designated as a PSSA in 1990 (Res. MEPC 44(30), 16.11.1990). Revised Guidelines in Resolution A. 982 (24 | 1991* | Particular Sensitive Sea Area: "A PSSA is an area that needs special protection through action by IMO because of its significance for recognized ecological, socio-economic, or scientific attributes where such attributes may be vulnerable to damage by international shipping activities. At the time of designation of a PSSA, an associated protective measure, which meets the requirements of the appropriate legal instrument establishing such measure, must have been approved or adopted by IMO to prevent, reduce, or eliminate the threat or identified vulnerability. | PSSA: Guidelines on designating a "particularly sensitive sea area" (PSSA) are contained in Resolution A.982(24) Revised guidelines for the identification and designation of Particularly Sensitive Sea Areas (PSSAs). Revised guidelines for the identification and designation of particularly sensitive sea areas. A 24/Res.982, 6 February 2006. | Water columnsurface | In EEZ, and territorial seas if partly in EEZ (no high seas application) | Shipping. When an area is approved as a particularly sensitive sea area, specific measures can be used to control the maritime activities in that area, such as routing measures, strict application of MARPOL discharge and equipment requirements for ships, such as oil tankers; and installation of Vessel Traffic Services (VTS) | IMO (MEPC) works with coastal states and flag states to identify and establish new PSSAs. A PSSA is an area that needs special protection through action by IMO because of its significance for recognized ecological, socio-economic, or scientific attributes where such attributes may be vulnerable to damage by international shipping activities. At the time of designation of a PSSA, an associated protective measure, which meets the requirements of the appropriate legal instrument establishing such measure, must have been approved or adopted by IMO to prevent, reduce, or eliminate the threat or identified vulnerability. | Charts and geographical coordinates indicating routing measures, areas to avoided, reporting duties. Each PSSA has its own particular protection regime. | MPA: There are seventeen PSSAs, located all over the world. Some are large, like the Western European Waters, and some protect tiny gems of nature like Malpelo Island in the Pacific Ocean. | [http://pssa.imo.org/#/pssas www.pssa.imo.org](http://pssa.imo.org/#/pssas ) |
| 4 | S | In | Marine Pollution Convention 1973/78 (MARPOL 773/78, as amended) | Annex I (persistent oil) and Annex II (HNS): 1983; Annex IV (sewage): 2003;Annex V (garbage): 1988 | Special areas. "2.1. MARPOL 73/78, in Annexes I, II and V, defines certain sea areas as Special Areas in relation to the type of pollution covered by each Annex. A Special Area is defined as "a sea area where for recognized technical reasons in relation to its oceanographic and ecological conditions and to the particular character of its traffic, the adoption of special mandatory methods for the prevention of sea pollution by oil, noxious liquid substances, or garbage, as applicable, is required." Under the Convention, these Special Areas are provided with a higher level of protection than other areas of the sea. 2.2 A Special Area may encompass the maritime zones of several States, or even an entire enclosed or semi-enclosed area. Special Area designation should be made on the basis of the criteria and characteristics listed in paragraphs 2.3 to 2.6 to avoid the proliferation of such areas." | Guidelines for the designation of Special Areas under MARPOL 73/78 adopted by resolution А.927(22). Resolution A.982(24) revokes annex 2 of resolution A.927(22) | Water columnsurface | Under national jurisdiction and beyond. Regional seas (TS, EEZ, high seas) plus Antarctica | Pollution from shipping. "Criteria for the designation of a Special Area: 2.3 The criteria which must be satisfied for an area to be given Special Area status are grouped into the following categories: i) oceanographic conditions; ii) ecological conditions; and iii) vessel traffic characteristics. Generally, information on each category should be provided in a proposal for designation. Additional information that does not fall within these categories may also be considered." | Treaty parties (coastal states on demand) within IMO (MEPC). About the procedures for the designation of a special area (3):  3.1 A proposal to designate a given sea area as a Special Area should be submitted to the Marine Environment Protection Committee (MEPC) for its consideration in accordance with the rules adopted by the IMO for submission of papers. 3.2 A proposal to designate a sea area as a Special Area should contain: 1) a draft amendment to MARPOL 73/78 as the formal basis for the designation; and 2) a background document setting forth all the relevant information to explain the need for the designation. | Charts, coordinates | • MARPOL Annex I (oil)  - Baltic Sea, Mediterranean Sea, Black Sea, Persian Gulf, Red Sea, Gulf of Aden, Antarctica and North Sea, N-W European Waters, Oman area of the Arabian Sea, Southern South-African waters • MARPOL Annex II (liquid chemicals)  - Baltic Sea and Black Sea / Antarctica (all discharges prohibited) • MARPOL Annex IV (sanitary waste)  - Baltic Sea • MARPOL Annex V (garbage)  - Baltic Sea, Black Sea, Mediterranean Sea, Persian Gulf, Red Sea, Caribbean (+ Gulf of Mexico), Antarctica and North Sea | The need to evaluate the effectiveness of Special Areas. IMO, 2016: "For Special Areas, the 2013 Guidelines for the designation of Special Areas under MARPOL 73/78 (resolution A.1087(28)) do not even give a recommendatory provision on reporting the environmental efficiency valuation of restrictive measures on discharges and emissions from ships in such sea areas." https://cil.nus.edu.sg/wp-content/uploads/2017/09/IMO-Doc.-MEPC-70-18-Report-Of-The-Marine-Environment-Protection-Committee-On-Its-Seventieth-Session.pdf |
| 5 | S | In | MARPOL 73/78, Annex VI (SECA + ECA) | 2005 | Air pollution from ships: SECA (Sulphur emission control areas), and ECA (emission control areas: Sulphur, nitrogen, PM) |  | Air, water column | Regional seas plus Antarctica plus coastal waters plus port areas (internal waters) | Low air emission zones for SOx, Nox and PM | Treaty parties (coastal states on demand) within IMO (MEPC) | Charts, coordinates | • SECAs: Baltic Sea, North Sea, North East Atlantic, 200 mile zone adjacent to North America, specific waters in the Pacific, Atlantic and Gulf coasts of the United States and Canada; selected waters in the Caribbean adjacent to Puerto Rico and the U.S. Virgin Islands. • Regional: e.g. EU ports, Chinese ECA zones: Yangtze River Delta, Shenzhen Port Area und Hong Kong harbor • North America is already an ECA. North Sea and Baltic Sea will become one in 2021 . | https://help.marinetraffic.com/hc/en-us/articles/214615067-ECA-Zones-Layer |
| 6 | S | In | Safety of Life at Sea Convention (SOLAS 74) (Area to be avoided) + 1993 General Provisions on Ships' routeing | 1980 (initially SOLAS V/8) | Areas to be avoided can be designated by the IMO under SOLAS regulation V/10 (Ships' Routeing); | IMO Resolution A.572(14) “General Provisions on Ships’ Routeing” (1985), as amended | Water column surface | Territorial sea and EEZ | Maritime safety, marine conservation, emergency preparedness and response | IMO MSC: In November 1997, the IMO Assembly adopted resolution A.858(20) by which it delegated to MSC the function of adopting traffic separation schemes, and routeing measures other than traffic separation schemes, including the designation and substitution of archipelagic sea lanes. | Navigation maps (ship routeing publication) plus coordinates | There are 9 ships’ routeing measures: traffic separation schemes (TSS), traffic lanes, separation zones, roundabouts, inshore traffic zones, recommended routes, deep-water routes, precautionary areas and areas to be avoided. Routeing measure along the Aleutian Islands: The purpose of this proposal is to reduce the risk of marine casualty and resulting pollution, protect the fragile and unique environment of the Aleutian Islands, and facilitate the ability to respond to maritime emergencies | http://www.nepia.com/media/258601/IMO-NCSR-2-3-5-Adopt-the-Establishment-of-Five-Areas-to-be-Avoided.pdf |
| 7 | S | In | International regulations for preventing collisions at sea (COLREG 1972), as frequently amended | 1977 | Traffic separation schemes and inshore traffic zones (rule 10) | IMO Ship's routeing | Water colum,surface | High sea and all therewith navigationable waters for seagoing vessels | Maritime safety | IMO MSC: In November 1997, the IMO Assembly adopted resolution A.858(20) by which it delegated to MSC the function of adopting traffic separation schemes, and routeing measures other than traffic separation schemes, including the designation and substitution of archipelagic sea lanes. | Navigation maps (ship routeing publication) plus coordinates | There are 9 ships’ routeing measures, and traffic separation schemes (TSS) is one of them. | http://www.imo.org/en/About/Conventions/ListOfConventions/Pages/COLREG.aspx |
| 8 | S | In | London (dumping) Convention and 1996 Protocol, as amended | 1975, 2006 | Zones for 'Carbon dioxide streams for carbon dioxide capture processes for sequestration' (Annex I.1.8: 2006); ocean fertilization and other marine geoengineering activities (art. 1. 5bis; art.6bis; Annexes 4 and 5: 2013) | Carbon sequestration areas under the seabed linked to former oil and gas wells. Marine geoengineering: placement site selection proposed by a party, taking into account effects for areas under jurisdiction of another party or beyond jurisdiction of any state (notification to that party and relevant IO + consultation) (Annex 5) | Water columnseabed subsoil | Territorial sea, continental shelf, EEZ, and high seas | pollution; scientific research | proposal party plus Meeting of the Parties (MOP, Protocol 1996) | Impact hypothesis; guidelines | (See refs.[1–3]) | http://www.imo.org/en/OurWork/Environment/LCLP/Pages/default.aspx |
| 9 | F | In | United Nations Fish Stocks Agreement (UNFSA) implementing art. 61, 63, 64 UNCLOS | 2001 | Art. 5 general principles (also apply in areas under national jurisdiction): - ensure long term sustainability of SFS and HMFS (e.g. tuna, marlin, swordfish, …) - assess impacts of fishing on target stocks and its ecosystem - adopt conservation and management measures - protect biodiversity  - prevent and eliminate overfishing - (…). Article 9. Subregional and regional fisheries management organizations and arrangements: 1. In establishing subregional or regional fisheries management organizations or in entering into subregional or regional fisheries management arrangements for straddling fish stocks and highly migratory fish stocks, States shall agree, inter alia, on: (a) the stocks to which conservation and management measures apply, taking into account the biological characteristics of the stocks concerned and the nature of the fisheries involved; (b) the area of application, taking into account article 7, paragraph 1, and the characteristics of the subregion or region, including socio-economic, geographical and environmental factors; (c) the relationship between the work of the new organization or arrangement and the role, objectives and operations of any relevant existing fisheries management organizations or arrangements; and (d) the mechanisms by which the organization or arrangement will obtain scientific advice and review the status of the stocks, including, where appropriate, the establishment of a scientific advisory body. | Conservation and management of straddling fish stocks and highly migratory fish stocks | Water columnsurface | EEZ, High seas | Fishery: conservation and management of living marine resources. Ensure the long-term conservation and optimum utilization of the fishery resources in the Convention Area, providing sustainable economic, environmental and social benefits. | Regional Fishery Management Organizations (RFMOs) | Maps and coordinates | See RFMOs | http://www.un.org/Depts/los/convention_agreements/convention_overview_fish_stocks.htm |
| 10 | F | R | NEAFC (North East Atlantic Fisheries Commission) | 1982 | Convention area (art. 1); Establishment of closed seasons and closed areas (art. 7.c) | Charts; maps. coordinates. Protection of Vulnerable Marine Ecosystems (VMEs) against bottom fisheries by making use of closed areas | Water columnsurfaceseabed | High seas, and EEZ if the party request so (art. 6) | Fisheries - seabed: protection, conservation (collective arrangement with OSPAR) | Measures are decided by the Parties which make up the Commission on the basis of scientific advice from an independent scientific body, the International Council for the Exploration of the Sea (ICES). The majority of these measures are decided at the Annual Meeting of the Commission held in November, but decisions can also be taken by postal vote throughout the year should the need arise. Article 5 of the NEAFC Convention: "The Commission shall, as appropriate, make recommendations concerning fisheries conducted beyond the areas under jurisdiction of Contracting Parties. Such recommendations shall be adopted by a qualified majority". | Areas closed for bottom fisheries to protect VMEs | 2004, 2009, 2014 (2014 in cooperation with OSPAR high sea MPAs | https://www.neafc.org/managing_fisheries/vmec linked to OSPAR high seas MPAs |
| 11 | F | R | SEAFO (South East Atlantic Fisheries Organization) | 2003 | Convention area (art. 4); closed fishing areas and periods (art. 6.8.b) | Charts; maps; coordinates. Protection of Vulnerable Marine Ecosystems (VMEs) against bottom fisheries by making use of closed areas | Water columnsurfaceseabed | High seas | Fisheries - seabed - conservation. Fish species in the Convention Area include sedentary / discrete and straddling species such as alfonsino, orange roughy, oreo dories, armourhead, sharks, deepwater hake and red crab. The inclusion of discrete high seas stocks takes the SEAFO Convention beyond the scope of the UNFSA. | Measures are decided by the Parties which make up the Commission. Conservation measure 30/15 on bottom fishing activities and VMEs in the SEAFO Convention Area. | 11 closed areas to bottom contact gears: approximately 505,000 km^2^. | 2015: SEAFO, Conservation Measure 30/15 on Bottom Fishing Activities and Vulnerable Marine Ecosystems in the SEAFO Convention Area, Adopted on 3rd December 2015 (available at http://www.seafo.org/) | http://www.seafo.org/ |
| 12 | F | R | SIOFA (South Indian Ocean Fisheries Agreement) | 2012 | Convention area (art. 3); closing areas (implicit in art. 6.1.d) | Charts; maps; coordinates; MPAs in future. Protection of Vulnerable Marine Ecosystems (VMEs) against bottom fishing by making use of closed areas or fisheries prohibition | Water columnsurfaceseabed | High seas | Fisheries - seabed - conservation and sustainable use. Covers fishery resources including fish, mollusks, crustaceans and other sedentary species within the area (excluding highly migratory species and sedentary species under jurisdiction of coastal states) | Measures are decided by the Parties which make up the Meeting of the Parties (MoP). CMM 2016/05 Pelagic driftnets and deepwater gillnets; CMM 2018/1 Interim Management of Bottom Fisheries, inter alia interim protected areas. | Prohibition of pelagic driftnets and deepwater gillnets in Convention area (CMM 2016/05); Scientific Committee to propose: Maps of VMEs; protocol for designation of MPAs; closing areas for particular gear. Interim measures for bottom fishing: cease bottom fishing in certain distances of VMEs. | 2016: CMM 2016/02; 2018: CMM 2018/01 | https://www.apsoi.org/ |
| 13 | F | R | SPRFMO (South Pacific Regional Fisheries Management Organization) | 2012 | Convention area (art. 5); closed areas, although not explicit mentioned in the Convention (see CM03-2018) | Charts; maps; coordinates. Protection of habitats, marine ecosystems, Vulnerable Marine Ecosystems (VMEs) against effects of fisheries. Pollution and waste from fishing vessels. | Water columnsurfaceseabed | High seas (plus under national jurisdiction of a party on its own initiative: Chile). | Fisheries - seabed - conservation and sustainable use of fisheries resources. Jack mackerel, chub mackerel, jumbo flying squid and other squid species, and to a much lesser degree deep-sea species often associated with seamounts and ridges. | Measures are decided by the Parties which make up the Commission. CMM 08-2013 Driftnets and gillnets; CMM 03-2018 Bottom Fishing; CMM 14b-2018 Exploratory Potting. | Prohibition of large-scale pelagic driftnets and all deepwater gillnets in Convention area (CMM 08-2013); Prohibition of bottom fishing at a certain distance of a VME, or in the entire area unless based on a bottom fishing footprint and impact assessment (CMM 03-2018); Boxed positions (longitude-latitude) for exploratory potting (lobster, crab) CMM 14b-2018. | 2013: CMM 08-2013; 2018: CMM 03-2018 and CMM 14b-2018. | https://www.sprfmo.int/ https://www.sprfmo.int/about/illustrative-map-of-sprfmo-area-2/ |
| 14 | F | R | NPFC (North Pacific Fisheries Commission) | 2015 | Area (art. 4); locations in which fishing activities shall not occur (art. 7.1.e.iii) | Coordinates, map, charts. Protection of habitats, marine ecosystems, Vulnerable Marine Ecosystems (VMEs) against effects of fisheries. Pollution and waste from fishing vessels. | Water columnsurfaceseabed | High seas (Bering Sea = excluded and high seas surrounded by the EEZ of 1 single state) | Fisheries - seabed - long-term conservation, sustainable use of fisheries resources and protection of marine ecosystems (VMEs). | Measures are decided by the Parties which make up the Commission. CMM 2018-5 Bottom fishing in the western part of the Convention area. | Bottom fisheries: In Convention area limit fishing effort. No expansion in western part if not occurring (seamounts). Not allow bottom fisheries below 1,500 m. Fishing some taxa is prohibited. Protection of certain cold water corals. Part of Koko seamount is closed for VME conservation. | 2018: CMM 2018-05 | https://www.npfc.int/ |
| 15 | F | R | General Fisheries Commission for the Mediterranean (GFCM) | 1952 (Council); 2004 (Commission) | Area: Mediterranean and Black Sea (art. 3); establishing fisheries restricted areas (FRAs) (art. 8.b.iv) | Coordinates, charts, maps, zones, MPA: fisheries restricted areas (FRAs) | Water columnsurfaceseabed | Territorial sea, continental shelf, EEZ, high seas | Fisheries conservation and the sustainable use of living marine resources as well as the sustainable development of aquaculture | Measures are decided by the Parties which make up the Commission. The Commission has the authority to adopt binding recommendations. | Fishery restricted areas (FRAs) | 9 FRAs: Rec. GFCM/29/2005/1 established a FRA below 1000 m prohibiting towed dredges and trawl nets; Rec. GFCM/30/2006/3 established 3 FRAs where deep-sea fisheries with towed dredges and bottom trawl nets are prohibited and MS have to protect these areas from the impact of any other activity; Rec GFCM/33/2009/1 FRA in Gulf of Lion limited number of fishery vessels allowed; Rec CM-GFCM/40/ 016/4 established 3 FRAs in Strait of Sicily where fishing with bottom trawl nets is prohibited and temporal fisheries closure in Gulf of Gabès; Rec GFCM/41/2017/3 FRA in the Jabula/Pomo Pit in the Adriatic Sea. | http://www.fao.org/gfcm/en/ |
| 16 | F | R | Common Fishery Policy (European waters) | 1957 (1958, Articles 38-43 of the Treaty on the Functioning of the European Union (TFEU).), 1970, 1983, 1992 (1993)  2002, 2013 | The Regulation (EU) no. 1380/2013, Art. 7: 2. “Technical measures may include, inter alia, the following: (…) (c) limitations or prohibitions on the use of certain fishing gears, and on fishing activities, in certain areas or periods; (d) requirements for fishing vessels to cease operating in a defined area for a defined minimum period in order to protect temporary aggregations of endangered species, spawning fish, fish below minimum conservation reference size, and other vulnerable marine resources; (e) specific measures to minimise the negative impact of fishing activities on marine biodiversity and marine ecosystems, including measures to avoid and reduce, as far as possible, unwanted catches.”  Art. 8, about the establishment of fish stock recovery areas: “1. The Union shall, while taking due account of existing conservation areas, endeavour to establish protected areas due to their biological sensitivity, including areas where there is clear evidence of heavy concentrations of fish below minimum conservation reference size and of spawning grounds. In such areas fishing activities may be restricted or prohibited in order to contribute to the conservation of living aquatic resources and marine ecosystems. The Union shall continue to give additional protection to existing biologically sensitive areas. 2. For those purposes, Member States shall identify, where possible, suitable areas which may form part of a coherent network and shall prepare, where appropriate, joint recommendations in accordance with Article 18(7) with a view to the Commission submitting a proposal in accordance with the Treaty. 3. The Commission may be empowered in a multiannual plan to establish such biologically sensitive protected areas. Article 18(1) to (6) shall apply. The Commission shall report regularly to the European Parliament and to the Council on protected areas.  Art. 9 about Multiannual plans: “Multiannual plans shall cover: (…), ii) in the case of mixed fisheries or where the dynamics of stocks relate to one another, fisheries exploiting several stocks in a relevant geographical area, taking into account knowledge about the interactions between fish stocks, fisheries and marine ecosystems.”  See also Recital 22, and | Territorial Use Right for Fishery (TURF), fishery closures, closures to certain gears, no-take areas, seasonal closures | Water columnsurfaceseabed | Territorial sea, EEZ, continental shelf, high seas  (The Regulation (EU) no. 1380/2013 specifies that the CFP applies (a) on the territory of Member States to which the Treaty applies; (b) in Union waters, including by fishing vessels flying the flag of, and registered in, third countries; (c) by Union fishing vessels outside Union waters; or (d) by nationals of Member States, without prejudice to the primary responsibility of the flag State. | The original objectives of the CFP were to preserve fish stocks, protect the marine environment, ensure the economic viability of European fleets and provide consumers with quality food. The 2002 reform added to these objectives the sustainable use of living aquatic resources in a balanced manner and from an environmental, economic and social point of view. The reform also specified that sustainability must be based on sound scientific advice and the precautionary principle.  The Regulation (EU) no. 1380/2013 states that the CFP scope is “the conservation of marine biological resources and the management of fisheries and fleets exploiting such resources”. | The Lisbon Treaty has given the European Parliament greater power to legislate, enabling it to help shape the Common Fisheries Policy and to supervise the rules that govern the activities of the EU’s fisheries and aquaculture sectors. Parliament has adopted a number of resolutions related to the need to reform the CFP.  The European Parliament (EP) is competent on fisheries legislation: the Lisbon Treaty provides for co-decision (the ordinary legislative procedure); EP is also responsible for EU membership of international fisheries conventions and the conclusion of agreements with third countries (co-decision with the Council).  About the implementation, the respective roles of Member States and of the European Commission are the following: i) Member States are responsible for the implementation of CFP rules on their territory and in their waters, and also by the vessels flying their flags operating outside these waters; ii) The Commission must ensure that Member States fulfil their obligations equally in terms of equity and effectiveness. It regularly draws up an evaluation report to Parliament and the Council on its action on the application of the CFP rules by Member States. | Territorial Use Right for Fishery (TURF), fishery closures, closures to certain gears, no-take areas, seasonal closures.  The Regulation (EU) no. 1380/2013, Art. 4, defines “(20) 'technical measure' means a measure that regulates the composition of catches by species and size and the impacts on components of the ecosystems resulting from fishing activities by establishing conditions for the use and structure of fishing gear and restrictions on access to fishing areas”. | For a review on the state of implementation of CFP see ref.[4] | https://www.europarl.europa.eu/factsheets/en/section/197/common-fisheries-policy |
| 17 | C | R | Vulnerable Marine Ecosystems, VME (Scientifically defined area under FAO - UN General Assembly (61/105, paragraph 90)) | 2006 | Vulnerability is related to the likelihood that a population, community, or habitat will experience substantial alteration from short-term or chronic disturbance, and the likelihood that it would recover and in what time frame. These are, in turn, related to the characteristics of the ecosystems themselves, especially biological and structural aspects. VME features may be physically or functionally fragile. The most vulnerable ecosystems are those that are both easily disturbed and very slow to recover, or may never recover. | VME closures, FRA and more | Any | Any | Management of impacts from deep sea fishery on vulnerable marine ecosystems | RFMOs | VME closed areas, FRA | See the database from FAO: http://www.fao.org/in-action/vulnerable-marine-ecosystems/vme-database/en/vme.html | http://www.fao.org/in-action/vulnerable-marine-ecosystems/en/ |
| 18 | C | R | CCAMLR (Convention on the Conservation of Antarctic Marine Living Resources) | 1982 | Antarctic marine living resources of the area south of 60° South latitude and to the Antarctic marine living resources of the area between that latitude and the Antarctic Convergence (line formed by coordinates) which form part of the Antarctic marine ecosystem (art. I). | Charts, maps, zones, MPA. Antarctic Specially Protected Area (ASPA) and Antarctic Specially Managed Area (ASMA). | Water columnsurfaceseabed | High seas and ABNJ of Antarctica | Fisheries: conservation of Antarctic marine living resources. Direct fishing prohibitions, closed areas, MPAs | Measures are decided by the Parties which make up the Commission. Conservation Measure 91-03 (2009) Southern Orkney Islands southern shelf MPA; Conservation Measure 91-04 (2011) General framework for the establishment of CCAMLR Marine Protected Areas; Conservation Measure 91-02 (2012) Protection of the values of Antarctic Specially Managed and Protected Areas; Conservation Measure 91-05 (2016) Ross Sea MPA from 1 December 2017; Conservation measures 32-02 (2017) prohibiting direct fishing on certain species in certain subareas. | Conservation Measure 91-03 (2009) Southern Orkney Islands southern shelf MPA in which all types of fishing is prohibited, except research fishing - no discharges or dumping of waste; Conservation Measure 91-04 (2011) General framework for the establishment of CCAMLR Marine Protected Areas; Conservation Measure 91-02 (2012) Protection of the values of Antarctic Specially Managed Areas (ASMA) and Antarctic Specially Protected Areas (ASPA) in which activities may be prohibited, restricted or managed in accordance with ASMA (10 ASPAs identified and 3 ASMAs); Conservation Measure 91-05 (2016) Ross Sea MPA from 1 December 2017; Ross Sea region MPA. Except as authorized under paragraphs 8, 9 and 21 fishing activities are prohibited within the Ross Sea MPA during 35 years. 9. Members may conduct directed fishing for Antarctic krill (*Euphausia superba*) in the Krill Research Zone and the Special Research Zone in accordance with Conservation Measure 51-04 and the specific objectives of the marine protected area in paragraph 3 of this conservation measure; Conservation measures 32-02 (2017) prohibiting direct fishing on certain species in certain subareas. | 2009: CM 91-03; 2001: CM 91-04; 2012 CM 91-02; 2016: CM 91-05; 2017 CM 32-02. | https://www.ccamlr.org/en/measure-91-05-2016 |
| 19 | C | In | ICRW (International Convention for the Regulation of the Whale (1946) | 1948 | Adoption of open and closed seasons and open and closed waters, including the designation of sanctuary areas (art. III-1) | Maps, coordinates. | Water column | EEZ, high seas | Environmental (species) protection: sanctuaries | International Whaling Commission (ICW) | Sanctuaries: areas with hunting prohibition (closed waters) | Two Sanctuaries are currently designated by the International Whaling Commission, both of which prohibit commercial whaling.  The first of these, the Indian Ocean Sanctuary, was established in 1979 and covers the whole of the Indian Ocean south to 55°S. The second was adopted in 1994 and covers the waters of the Southern Ocean around Antarctica. The precise co-ordinates are recorded in the Schedule at paragraphs 7.(a) and 7.(b) | https://iwc.int/sanctuaries |
| 20 | C | In | 1971 Convention on Wetlands of International Importance Especially as Waterfowl Habitat, amended by the Protocol 1982 and the Amendments of 1987 (Ramsar Convention) | 1975 | Framework for national action and international cooperation for the conservation and wise use of wetlands and their resources. Art. 1 "wetlands are areas of marsh, fen, peatland or water, whether natural or artificial, permanent or temporary, with water that is static or flowing, fresh, brackish or salt, including areas of marine water the depth of which at low tide does not exceed six meter". Art. 2.1. Each Contracting Party shall designate suitable wetlands within its territory for inclusion in a List of Wetlands of International Importance (...) The boundaries of each wetland shall be precisely described and also delimited on a map and they may incorporate riparian and coastal zones adjacent to the wetlands, and islands or bodies of marine water deeper than six meters at low tide lying within the wetlands, especially where these have importance as waterfowl habitat.” | MPAs: maps | Surfacewater columncoastal seabed | Coastal (territorial sea) and inland waters | Environmental conservation | States (contracting parties) | MPAs | The world’s first Site was the Cobourg Peninsula in Australia, designated in 1974. Number of Ramsar Sites: 2,341. Total surface of designated sites: 252,479,417 ha. | https://www.ramsar.org/sites-countries/ramsar-sites-around-the-world |
| 21 | C | In | Convention on Biological Divesity (CBD) | 1993 | Art. 8 about in-situ conservation: "Each Contracting Party shall, as far as possible and as appropriate: (a) Establish a system of protected areas or areas where special measures need to be taken to conserve biological diversity; (b) Develop, where necessary, guidelines for the selection, establishment and management of protected areas or areas where special measures need to be taken to conserve biological diversity; (...)" Art. 10 Article 10. Sustainable Use of Components of Biological Diversity "Each Contracting Party shall, as far as possible and as appropriate: (...) (d) Support local populations to develop and implement remedial action in degraded areas where biological diversity has been reduced | MPAs | Under seabedseabed water columnsurface | Territorial sea, EEZ, High seas, continental shelf.  About ABNJ: Art. 8 does not explicitly refer to ABNJ, but art. 4 (b) and art. 5 do. There are divergent opinions on the fact that art. 8 applies to ABNJ (F. Maes, personal communication). Nevertheless, art. 4 (a)(b) and art. 5 apply to all marine zones. | Environmental conservation | No, sovereign states | MPA: cooperation (art.5 CBD), strategies, plans or programmes (art.6 CBD), establish a system of protected areas or areas where special measures need to be taken to conserve biological diversity (Art.8a), develop guidelines for the selection, establishment and management of such areas (Art.8b), restoration (art.8f) etc. | About the mechanisms of implementation see: https://www.cbd.int/mechanisms/ | https://www.cbd.int/convention/articles/default.shtml?a=cbd-08, https://www.cbd.int/convention/articles/default.shtml?a=cbd-10 |
| 22 | C | In | Aichi Biodiversity Targets | 2010 | Strategic Goal C: To improve the status of biodiversity by safeguarding ecosystems, species and genetic diversity, Target 11: By 2020, at least 17 per cent of terrestrial and inland water, and 10 per cent of coastal and marine areas, especially areas of particular importance for biodiversity and ecosystem services, are conserved through effectively and equitably managed, ecologically representative and well connected systems of protected areas and other effective area-based conservation measures, and integrated into the wider landscapes and seascapes. | target 11 - 10% area conservation (objective) | Seabedwater columnsurface | Territorial sea, EEZ, High seas, continental shelf, ABNJ | Environmental conservation | No, sovereign states | MPA: systems of protected areas and other effective area-based conservation measures | The status of the implementation of the Aichi targets are available at https://chm.cbd.int/search/reporting-map?filter=AICHI-TARGET-11 | https://www.cbd.int/sp/targets/default.shtml |
| 23 | C | In | CBD COP Decisions |  | MPAs are one of the essential tools and approaches in the conservation and sustainable use of marine and coastal biodiversity (Decision VII/5 Marine and coastal biological diversity, UNEP/CBD/COP7/21,) as well as the biodiversity beyond the limits of national jurisdiction (Decision VIII/24 Protected area, UNEP/CBD/COP8/31); CBD Decision VII/28 to establish by 2012 a representative system of marine and coastal protected areas; Increase efforts to apply marine spatial planning tools, as appropriate, in accordance with Parties ‘national planning and strategies, for better integration of conservation objectives in marine and other sectoral development programmes, and in overall plans for economic development’ (CBD Decision X/29); In 2012, COP 11 adopted for the first time, a decision on MSP (COP, 2012, Decision XI/18, section c). This Decision was a start to bring MSP under the universal attention of the CBD Parties, other governments and competent organizations by disseminating MSP awareness-raising material and by initiating the development of a web-based information sharing system on MSP; COP Decision XII/22. Marine and coastal biodiversity: ecologically or biologically significant marine areas (EBSAs) plus Description of areas meeting the EBSA criteria: In 2010, COP 10 noted that the application of the EBSA criteria is a scientific and technical exercise, that areas found to meet the criteria may require enhanced conservation and management measures, and that this can be achieved through a variety of means, including marine protected areas and impact assessments. The COP further noted that the application of the EBSA criteria is an open and evolving process that should be continued to allow ongoing improvement and updating as improved scientific and technical information becomes available in each region; Decision XII/23 Recognizing that marine spatial planning is a useful tool for applying the ecosystem approach to marine and coastal management, and considering the challenges associated with its implementation, requests the Executive Secretary and invites relevant organizations to advance their work on enhancing methods and tools, including monitoring measures, for marine spatial planning; Decision XIII/9. Marine spatial planning and training initiatives; Decision XIII/12. Marine and coastal biodiversity: ecologically or biologically significant marine areas; Decision 14/8. Protected areas and other effective area-based conservation measures. | MPAs and other effective area-based conservation measures, ecological corridors, buffer zones, trans-boundary protected areas (TBPAs), MSP, description of Ecologically or Biologically Significant marine Areas (EBSAs) on the basis of coordinates. CBD scientific criteria for ecologically or biologically significant areas (EBSAs) (annex I, Decision IX/20) | Seabedwater columnsurface | Territorial sea, EEZ, High seas, continental shelf, ABNJ | Environmental conservation. MSP is seen as a tool for long-term conservation, management and sustainable use of marine resources and coastal habitats, and to effectively manage MPAs | No, sovereign states. Regional Workshops to identify EBSAs to be adopted by States during CBD COPs. | MPA: COP Decision XII/22 on EBSAs: 39 areas meeting the EBSA criteria in the Southern Indian Ocean; 21 areas meeting the EBSA Criteria in the Eastern Tropical and Temperate Pacific; 20 areas meeting the EBSA criteria in the North Pacific; 45 areas meeting the EBSA criteria in the South-Eastern Atlantic; 11 areas meeting the EBSA criteria in the Arctic; 7 areas meeting the EBSA Criteria in the North-West Atlantic; 17 areas meeting the EBSA criteria in the Mediterranean. COP Decision XIII/12 on EBSAs: 10 areas meeting the EBSA criteria in the North-East Indian Ocean; 30 areas meeting the EBSA Criteria in the North-West Indian Ocean and Adjacent Gulf Areas; 36 areas meeting the EBSA criteria in the East Asian Seas; COP Decision 14/8 Voluntary guidance on the integration of protected areas and other effective area-based conservation measures into wider land- and seascapes and mainstreaming across sectors to contribute, inter alia, to the sustainable development goals; COP Decision 14/9. Marine and coastal biodiversity: ecologically or biologically significant marine areas: 33 areas meeting the EBSA criteria in the Black Sea and the Caspian Sea; 9 areas meeting the EBSA criteria in the Baltic Sea. The new EBSAs were identified in Helsinki earlier in February 2018 during the Baltic EBSA workshop convened by the UN Secretariat of the Convention on Biological Diversity (CBD) in collaboration with HELCOM, with financial support from Finland and Sweden. | 2004, 2010, 2012, 2014, 2016, 2018: 2004, Decision VII/5; 2004, Decision VII/28; 2010 Decision X/29. Study on `Marine Spatial Planning in the context of the Convention on Biological Diversity` (Secretariat CBD, 2012); 2012, Decision XI/18; SBSTTA (2012a), Synthesis document on the experience and use of marine spatial planning, UNEP/CBD/SBSTTA/16/INF/18, at www.cbd.int/sbstta16/documents/; 2014, COP Decision XII/22 on EBSAs; 2014 COP Decision XII/23; 2016 COP Decision XIII/9; 2016 COP Decision XIII/12; 2018 COP Decision 14/8; 2018 COP Decision 14/9. | http:/www.cbd.int/doc/decisions/COP-07-dec-en.pdf http:/www.cbd.int/doc/decisions/COP-08-dec-en.pdf) SBSTTA (2012b), Marine Biodiversity: Marine Spatial Planning and Voluntary Guidelines for the Consideration of Biodiversity in Environmental Impact Assessments and Strategic Environmental Assessments in Marine and Coastal Areas, UNEP/CBD/SBSTTA/16/7, at www.cbd.int/sbstta16/documents/.  Secretariat of the Convention on Biological Diversity and the Scientific and Technical Advisory Panel – GEF (2012), Marine Spatial Planning in the Context of the Convention on Biological Diversity. A study carried out in response to CBS COP 10 decision X/29, Montreal, Technical Series N. 68; SBSTTA (2012a), Synthesis document on the experience and use of marine spatial planning, UNEP/CBD/SBSTTA/16/INF/18, at www.cbd.int/sbstta16/documents/; https://www.cbd.int/ebsa/ebsas |
| 24 | C | R | 1979 Convention on the Conservation of European Wildlife and Natural Habitats (Bern Convention) | 1982 | The Parties undertake to take all appropriate measures to ensure the conservation of the habitats of the wild flora and fauna species. Such measures should be included in the Parties planning and development policies and pollution control, with particular attention to the conservation of wild flora and fauna. | PA (protected areas). Recommendation No. 16 (1989) on Areas of Special Conservation Interest (ASCI) and Recommendation No. 25 (1991) on the conservation of natural areas outside protected areas proper. | Coastal area (mainly land) | Land side coasts | Environmental conservation of specific marine species, but limited (e.g. marine turtles) | States party | Plans of designates areas | No examples in marine areas known | https://www.coe.int/en/web/bern-convention/ |
| 25 | C | R | European Union | 2005 - 2008 | Thematic Strategy for the Marine Environment: relies on marine regions for an ecosystem-based approach, most of them delimitated on the basis of European regional seas, such as the Baltic Sea, the North Sea, Mediterranean Sea, Black Sea and identified sub areas (e.g., Iberian coastal sea, Celtic-Biscay Shelf) in the North East Atlantic Ocean. The main component of the Marine Strategy is the Marine Strategy Framework Directive to achieve good environmental status of the marine environment by 2020 at latest. | Marine regions: Communication from the Commission to the Council and the European Parliament. Thematic Strategy on the Protection and Conservation of the Marine Environment. COM(2005)504. Brussels, 24.10.2005. Directive 2008/56/EC of the European Parliament and of the Council of 17 June 2008 establishing a framework for community action in the field of marine environmental policy (Marine Strategy Framework Directive). The Directive aims to achieve Good Environmental Status (GES) of the EU's marine waters by 2020. | Seabedwater column surface | Territorial sea, EEZ, High seas, continental shelf | Environmental conservation and protection; pollution | Member States (MS) | Coordinates, maps | The technical reports per country on the MSFD Plans of measures are available at https://ec.europa.eu/environment/marine/eu-coast-and-marine-policy/implementation/reports_en.htm. In the Annex 2 of the Report from the European Commission to the European Parliament and the Council  on the progress in establishing marine protected areas[5] (as required by Article 21 of the Marine Strategy Framework Directive 2008/56/EC) the state of MPA coverage is assessed for the year 2012. | http://ec.europa.eu/environment/marine/eu-coast-and-marine-policy/marine-strategy-framework-directive/index_en.htm |
| 26 | C | R | European Union | 1979; 2009 | Birds Directive. Birds Directive 79/409/EEC in April 1979 is the oldest piece of EU legislation on the environment and one of its cornerstones. Amended in 2009, it became Directive 2009/147/EC. MS must create protected areas (art. 3.2.a) and biotopes (art. 3.2.d), classified as Special Protection Areas (SPAs) (art. 4.1). | Special Protection Areas (SPAs) : Directive 2009/147/EC of the European Parliament and of the Council of 30 November 2009 on the conservation of wild birds. | Surface water | Land, internal water and territorial sea | Environmental conservation: birds | MS must designate Special Protection Areas (SPAs); control by the EU Commission | MPA: coordinates, maps | The ORNIS Committee assists the Commission in the implementation of the Birds Directive. | http://ec.europa.eu/environment/nature/legislation/birdsdirective/index_en.htm |
| 27(a) | C | R | European Union | 1992 | Habitat Directive. Directive 92/43/EEC of 21 May 1992 on the conservation of natural habitats and of wild fauna and flora.   Set up an ecological network of Special Areas of Conservation (SACs) under Natura 2000 (art. 3.1.; art. 6). Core areas of habitats shall be designated by MS + the Commission as Sites of Community Importance (art. 1.k, art. 8.1).   For species and sub- species listed in Annex IV (including many that are also listed in annex II) a strict protection regime must be applied across their entire natural range within the EU, both within and outside Natura 2000 sites | MPAs, called SACs (Special Areas of Conservation and Sites of Community Importance (SCIs): Directive 92/43/EEC of 21 May 1992 on the conservation of natural habitats and of wild fauna and flora. | Seabedwater columnsurface | Land, internal water, territorial sea, continental shelf, EEZ | Environmental conservation: habitats and wild fauna and flora (ensure the survival of Europe’s most endangered and vulnerable species, e.g. cetaceans) | MS with control by the EU Commission | MPA: coordinates, maps | The Habitats Committee assists the Commission in the implementation of the ‘Habitats’ Directive. | https://ec.europa.eu/environment/nature/legislation/habitatsdirective/index_en.htm |
| 27  (b) | C | R | European Union | 2000 | Natura 2000 is a network of core breeding and resting sites for rare and threatened species, and some rare natural habitat types which are protected in their own right. It stretches across all 28 EU countries, both on land and at sea. The aim of the network is to ensure the long-term survival of Europe's most valuable and threatened species and habitats, listed under both the Birds Directive and the Habitats Directive. Stretching over almost 6 % of the EU marine territory, it is the largest coordinated network of protected areas in the world. It offers a haven to Europe's most valuable and threatened species and habitats. | MPAs - Sites of Community Importance (SCIs): ecological network of protected areas. The Natura 2000 Viewer is an on-line tool that presents all Natura 2000 sites, provides key information on species and habitats for which each site has been designated, data on their estimated population size, conservation status and allows for various searches. | Seabed water columnsurface | Land, internal water, territorial sea, continental shelf, EEZ | Environmental conservation: habitats and wild fauna and flora | MS with control by the EU Commission | MPA: GIS, maps | see sites at http://ec.europa.eu/environment/nature/natura2000/index_en.htm | http://natura2000.eea.europa.eu/# http://ec.europa.eu/environment/nature/natura2000/faq_en.htm |
| 28 | C | R | Mediterranean: Barcelona Convention (1976 amended in 1995 extending the coverage to internal waters + coastal areas); Protocol concerning Specially Protected Areas and Biological Diversity in the Mediterranean (SPA 1982 amended 1995) and the 1996 Annexes, amended in 2009, 2012, 2013; ICZM Protocol 2008. See for fisheries: GFCM | 2009 | SPA Protocol, Section 1 on Specially Protected Areas, Section 2 on SPA of Mediterranean Importance. ICZM Protocol: "integrated coastal zone management" means a dynamic process for the sustainable management and use of coastal zones, taking into account at the same time the fragility of coastal ecosystems and landscapes, the diversity of activities and uses, their interactions, the maritime orientation of certain activities and uses and their impact on both the marine and land parts. | SPA and ICZM (Protocols) | Seabedsubsoil, water columnsurface | Land, internal water and territorial sea, high sea | Environmental conservation, land-sea interaction | Contracting parties: 22 Mediterranean riparian countries | MPA ; ICZM | See initiatives and the state of implementation at https://web.unep.org/unepmap/ | http://web.unep.org/unepmap/1-barcelona-convention-and-amendments |
| 29 | C | R | ACCOBAMS (Agreement on the Conservation of Cetaceans of the Black Sea, Mediterranean Sea and Contiguous Atlantic Area (1996) | 2001 | Art. 1. geographic scope of the Agreement: maritime waters of Black Sea, Mediterranean and the contiguous Atlantic Area (by Resolution in 2010 expanded to the EEZs of Spain and Portugal in het Atlantic Ocean); art. II.1. Parties shall take coordinated measures to achieve and maintain conservation status for cetaceans. To this end, Parties shall prohibit and take all necessary measures to eliminate; where this is not already done, any deliberate taking of cetaceans and shall cooperate to create and maintain a network of specially protected areas to conserve cetaceans; Annex 2, 3. Habitat protection: "Parties shall endeavor to establish and manage specially protected areas for cetaceans corresponding to the area which serve as habitats for cetaceans and/or which provide important food resources for them. Such specially protected areas should be established within the framework of the Regional Seas Conventions (OSPAR, Barcelona and Bucharest Conventions), or within the framework of other appropriate instruments"; ACCOBAMS-MOP3/2007/Res. 3.22, Annex 1 "Criteria for the selection of protected areas" | Specially protected areas (SPA) | Water column | Internal waters connecting maritime waters, territorial seas, EEZ, high sea | Environmental conservation | Reference to other conventions; Meeting of the ACCOBAMS Parties (MOP) | MPA | ACCOBAMS-MOP3/2007/Res. 3.22 establishing 9 SPA for the common dolphin and other cetaceans, 3 SPA for Black Sea cetaceans, 3 SPA for bottlenose dolphins, 1 SPA for sperm whales and 2 SPA for various cetacean species; ACCOBAMS-MOP4/2010/Res. 4.15 adds 1 SPA for the common dolphin and other cetaceans, 2 SPA for bottlenose dolphins and 1 SPA for various cetacean species. | http://www.accobams.org/conservations-action/protected-areas/ |
| 30 | C | R | ASCOBANS (Agreement on the Conservation of Small Cetaceans in the Baltic, North East Atlantic, Irish and North Sea (1992, amended in 2003) | 1994; 2008 | Art. 1.2.b. geographic coverage of the Agreement (coordinates) | No reference to special areas in the Agreement | Water column | Territorial sea, EEZ | Environmental conservation | No | Parties refer to EU Habitats Directive (SAC), EU Natura 2000, OSPAR (MPA) and Helcom (BSPA) for protected areas | No examples | http://www.accobams.org/ |
| 31 | C | R | OSPAR Convention (North East Atlantic), Paris 1992. Annex V: MPAs | 1992, 2000, 2003 | Within OSPAR, MPAs are understood as areas for which protective, conservation, restorative or precautionary measures have been instituted for the purpose of protecting and conserving species, habitats, ecosystems or ecological processes of the marine environment. Art. 3.1. b.ii, Annex V, OSPAR Convention: it shall be the duty of the Commission "... to develop means, consistent with international law, for instituting protective, conservation, restorative or precautionary measures related to specific areas or sites related to particular species or habitats". Recom. 2002/3: "Marine protected area” means an area within the maritime area for which protective, conservation, restorative or precautionary measures, consistent with international law have been instituted for the purpose of protecting and conserving species, habitats, ecosystems or ecological processes of the marine environment. The Ministerial meeting of the OSPARCOM in Bergen adopted the Bergen Statement (2010), which mainly focused on the contribution by OSPAR to the implementation of the EU Marine Strategy Framework Directive (MSFD) to protect species and habitats, including MPAs, within and beyond national jurisdiction, and to focus on various sources of pollution and adverse impacts of human activities. | MPAs: Recommendation 2003/3 on a network of marine protected areas, amended by Recommendation 2010/2 | Seabed water columnsurface | Mainly territorial sea and EEZ, but also High seas and continental shelfs. | Environmental conservation | Nomination to the OSPAR list by Contracting Parties. | MPA: OSPAR network of MPAS in national waters and ABNJ: coordinates, charts, maps | 2003; There are 465 MPAs, covering 6.3% of the North-East Atlantic (2018). For example: Protection and conservation of seamounts (OSPAR Rec. 2014/9); Protection and conservation of carbonate mounds in region V (OSPAR Decision 2014/10); Protection and conservation of hydrothermal vents/fields occurring on oceanic ridges in Region V (OSPAR Rec. 2014/11); There are 10 OSPAR MPAs BNJ. | https://www.ospar.org/work-areas/bdc/marine-protected-areas |
| 32 | C | R | OSPAR MPA high seas + NEFAC (closed bottom fisheries) | 2010 (2014) | For ABNJ OSPAR has developed specific instruments for MPAs (designation through legally binding OSPAR Decisions; and management through OSPAR Recommendations); see also NEFAC | MPAs | Seabedwater column | High seas | Environmental conservation | Parties within OSPARCOM | MPA | Since 2007, OSPAR has been considering proposals for several sites in ABNJ as possible MPAs. The 2010 OSPAR Ministerial Meeting took the significant step of adopting OSPAR Decisions establishing six MPAs in ABNJ and OSPAR Recommendations on their initial management. This was followed, in 2012 by the designation of a seventh MPA in ABNJ, including a Recommendation for management. | https://www.ospar.org/work-areas/bdc/marine-protected-areas/mpas-in-areas-beyond-national-jurisdiction |
| 33 | C | R | HELCOM (Baltic Sea), Helsinki Convention 1992: MPAs | 1992 - 1994 | Art. 15 Helsinki Convention 1992; HELCOM Recommendation 15/5 "System of coastal and marine Baltic Sea Protected Areas (BSPA)(1994) propose 64 areas; HELCOM Recommendation 35/1 "System of coastal and marine Baltic Sea protected areas (HELCOM MPA​s)(2014) supersedes HELCOM 15/5 | MPAs; BSPA | Seabed water columnsurface | Territorial sea, EEZ, continental shelfs | Environmental conservation | Coastal state | MPA; BSPA: coordinates, maps | [1994; 176 HELCOM MPAs in the Baltic Sea (2018); 9 ecologically unique marine areas in the Baltic Sea are included in a global registry during the UN Biodiversity Conference held in November 2018. These Ecologically or Biologically Significant Marine Areas (EBSAs) cover 23 percent of the Baltic Sea waters. Five are transboundary areas, spanning over waters of two or more countries.](https://www.cbd.int/conferences/2018) | http://www.helcom.fi/Lists/Publications/BSEP105.pdf http://www.helcom.fi/news/Pages/New-EBSAs-in-the-Baltic-Sea.aspx |
| 34 | C | R | Black Sea - Bucharest Convention on the Protection of the Black Sea Against Pollution (1992). Black Sea Biodiversity and Landscape Conservation Protocol Against (2002); Protocol on the Protection of the Marine Environment of the Black Sea from Land-Based Sources and Activities (2009) [entry into force pending] | 1994; 2011 (Protocol 2002) | Art. I Geographical coverage (Black Sea); Art. 3 Protocol: The area to which this Protocol applies shall be the area of the Black Sea to the north of capes Kalagra and Dalyan, the waters, sea bed, subsoil up to the fresh water limits. It also includes: · The Sea of Azov as a part of the Black Sea Basin highly important for the biodiversity and landscape conservation; · The coastal zone designated by each Contracting Party, including wetlands. | MPAs (art. 4 Protocol 2002 and Annex I - procedure). ICZM (art. 7, Protocol 2002 and art. 4, Protocol 2009) + ICZM Guidelines | Seabedwater colum  surfacesubsoil | Coastal zones (including wetlands) proposed by contracting parties; territorial sea and EEZ | Environmental conservation | Coastal state (parties) | MPA, ICZM | 2002: some 125 protected areas have been designated bordering the Black Sea coast. These vary in size from tiny scientific reserves of 1 ha up to the newly designated Zernov's Phyllophora Field in the northwest shelf of Ukraine (402,500 ha). However, in many cases, it is not clear from the designation data whether or not any coastal zones, let alone sub-shore marine waters, are covered by the protected area. | http://www.blacksea-commission.org/_convention-protocols-biodiversity.asp http://www.blackseascene.net/content/content.asp?menu=0180029_000000 |
| 35 | C | R | Convention for Cooperation in the Protection, Management and Development of the Marine and Coastal Environment of the Atlantic Coast of the West, Central and Southern Africa Region (Abidjan Convention, 1981) | 1984 | Art. 1 Geographical coverage Convention; art. 11 Specially protected areas; Decision CP. 9/12. Development of a Marine Protected Areas Protocol (2011) | SPA, MPA | Seabedwater column | Inland waters, territorial sea (though at the Fifth Meeting of the Contracting Parties in March 2000 it was proposed to include EEZ, it was not approved by the parties) | Environmental conservation | Coastal states (parties) | MPA | Recommendation Experts Segment " 9. States are encouraged to contribute to the establishment of Marine Protected Area (MPA) networks, designed in the framework of the joint IUCN-Abidjan Convention initiative, in order to strengthen regional networks of MPAs such as the Regional Network of Marine Protected Areas in West Africa (RAMPAO) and the Regional Network of Protected Areas in Central Africa (RAPAC).": UNEP(DEPI)/WACAF/COP. 10/12 (2012) | https://abidjanconvention.org/ |
| 36 | C | R | RAMPAO (Réseau régional des Aires Marines Protégées en Afrique de L'Ouest) - Regional, strategy for the West African ecoregion | 2010 | In 2002, a Regional Strategy for MPAs was developed by the various groups of stakeholders involved. In 2003, this regional strategy secured through the signing of a general policy statement by ten ministers in charge of environment, protected areas and fishing in six countries (Cape Verde, the Gambia, Guinea Bissau, Guinea, Mauritania and Senegal). The RAMPAO Network was officially set up during its constituent assembly held in Praia, Cape Verde in 2007. Then in 2010, the seven States formally recognized the Network through the signing of a declaration by 15 ministers, which enabled the Network to set up its institutional credibility and promote its development as a contribution to the implementation of the countries’ international undertakings. | MPA | Seabedwater column | Coastal water, inland waters | Environmental protection, mangroves, wetlands | Coastal states (parties) | MPA |  | www.rampao.org |
| 37 | C | R | Convention for the Protection of the Marine Environment and Coastal Area of the South-East Pacific (Lima, 1981); Protocol for the Conservation and Management of Protected Marine and Coastal Areas of the South-East Pacific (Paipa, 1989) |  | Art. II Protocol: "Parties shall establish areas under their protection in the form of parks, reserves, flora and fauna sanctuaries and other such areas. In these areas integration management shall be established on the basis of studies and inventories of their resources, with a view to ensuring their sustained development, and any activity liable to have adverse effects on the ecosystem, flora and fauna or their habitat, shall be prohibited". Art; III Information on PAs, Art. IV Common criteria, Art. V Regulation of activities, Art. 6 Buffer zones, Art. 7 Prevention and reduction of pollution of PA | PAs (protected areas). | Seabedwater column | Coastal zone, territorial sea, continental shelf, EEZ | Environmental protection | Coastal states (parties) | MPA | For a description of the state of the network of MPAs at year 2010 see ref.[6] | http://www.cpps-int.org/index.php/principal |
| 38 | C | R | Convention for the Protection and Development of the Marine Environment in the Wider Caribbean Region (WCR) (Cartagena, 1983); Protocol Concerning Specially Protected Areas and Wildlife (SPAW) in the Wider Caribbean Region (SPAW Protocol, 1990) | 1986; 2000 | Art. 2.1 Convention area, art. 10 Specially protected areas; SPAW Protocol: art. 1.c def Wider Caribbean region, art. 3 duty to protect, preserve and manage PAs, art. 4 establishment of PAs, art. 5 protection measures, art. 6 planning and management of PAs, art. 7 cooperation, art. 8 establishment of buffer zones, art. 10-11 flora and fauna protection + annexes (list of protected species revised in 2016) | SPA | Seabedwater column | Inland water (up to fresh water limit), coastal water (including watersheds) territorial sea, EEZ | Environmental protection | Proposal coastal states, review WG evaluation PAs, approval parties to the Protocol (COP) | MPA | 32 protected areas listed (see UNEP(DEPI)/CAR WG 40/4, 21 November 2018). In 2012: Belize: Hol Chan Marine Reserve and Glover’s Reef Marine Reserve; Cuba: Guanahacabibes National Park; Colombia: Sanctuary Cienaga Grande de Santa Marta and Regional Seaflower Marine Protected Area in San Andrés and Providencia Archipelago; France: Grand Connétable Island Natural Reserve (French Guyana), National Park of Guadeloupe, St Martin National Reserve and Lagoon Ponds, Petite-Terre National Reserve (Guadeloupe), and Agoa Sanctuary (FWI); the Caribbean Netherlands: Bonaire National Marine Park, St Eustatius National Park, the Quill/Boven National Park and Saba Bank National Park; United States of America: Florida Key National Marine Sanctuary, Dry Tortugas National Park, Everglades National Park in Florida, and Flower Garden Banks National Marine Sanctuary in Texas. In 2014: Colombia: Regional Natural Park of Wetlands between the rivers León and Suriquí; Kingdom of the Netherlands: Saba National Marine Park, St Eustatius National Marine Park, and Man O War Shoal Marine Park (Sint Maarten) ; France: Reserve Etang des Salines and Reserve Versants Nord de la Montagne Pelée in Martinique; Belize: Port Honduras Marine Reserve; Dominican Republic: La Caleta Submarine Park, National Park Jaragua, National Park Haitises, and National Park Sierra de Bahoruco; Saint Vincent and the Grenadines: Tobago Cay Marine Park. in Grenada: Molinière-Beauséjour Reserve. In 2017: Cuba: National Parc Cayos de San Felipe. | http://www.spaw-palisting.org/ http://www.car-spaw-rac.org/?Cartagena-and-SPAW-introduction,50 http://www.car-spaw-rac.org/?Protected-Areas-listed-under-the,715 |
| 39 | C | R | Regional Convention for the Conservation of the Red Sea and Gulf of Aden Area (PERSGA) - Jeddah (1982). Protocol Concerning the Conservation of Biological Diversity and the Establishment of Network of Protected Areas (2005) | 1985; | Art. II Geographical coverage Convention (Red Sea, Gulf of Aqaba, Gulf of Suea, Suez Canal, Gulf of Aden); Protocol: Art. 2.11 (def. Protected area: "geographically defined coastal and marine areas that are designated or regulated and managed to achieve specific conservation objective), Art. 4.2 duty to protect and conserve, Part 3 protection of marine and coastal areas (art. 9 - 13), Part 4 ICZM (art. 14) | (M)PAs (art. 9 establishment, art. 10 inclusion in PERSGA list, art. 11 management of PAs, art. 12 areas of special importance, art. 13 management of special habitats); ICZM (art. 14 Integrated Coastal Areas Management) | Seabedwater column | Inland water, coastal water, territorial sea | Environmental conservation | Coastal state | MPA, ICZM | Marine protected areas (MPAs) have been established in many parts of the Red Sea and Gulf of Aden. Of particular importance is the need to conserve areas that represent the range of the region’s unique habitats and biodiversity and to support national development. This has been initiated through the integration of 12 MPAs from throughout the region into a Regional Network of MPAs for the Red Sea and Gulf of Aden. These 12 MPAs are: Iles des Sept Frères and Ras Siyan (Djibouti); Ras Mohammed National Park; Giftun Islands and Straits of Gubal (Egypt); Aqaba coral reefs (Jordan); Straits of Tiran; Wajh Bank, Sharm Habban and Sharm Munaybirah; Farasan Islands (Saudi Arabia); Aibat and Saad ad-Din Islands, Saba Wanak (Somalia); Sanganeb Marine National Park; Mukkawar Island and Dungonab Bay (Sudan); Socotra Islands; Belhaf and Bir Ali area (Yemen). | http://www.persga.org/inner.php?id=109 http://www.persga.org/inner.php?id=110 |
| 40 | C | R | Framework Convention for the Protection of the Marine Environment of the Caspian Sea (Tehran, 2003); Protocol for the Conservation of Biological Diversity (Ashgabat, 2014) | 2006 (Protocol not yet into force) | Caspian Sea (art. 3, Convention), art. 15 Coastal zone management; Protocol: art. 2.c protect and conserve areas that best represent the high rate of species, special habitats, ecological systems, …, Art. 5.d. ... establishment of PAs, Art. 9 designation of protected areas, Art. 10 management of PAs, Art. 11 procedures for the establishment and listing of PAs, Art. 12 conservation of biological diversity in the framework of coastal zone management | PA; ICZM | Water column | Coastal water, territorial sea | Environmental conservation | Coastal state | MPA, ICZM |  | http://www.tehranconvention.org/ |
| 41 | MSP | R | OSPAR (North East Atlantic): MSP | 2002 | MSP was triggered by the Ministers of Environment of the countries bordering the North Sea at their 5th International Conference on the Protection of the North Sea (Bergen Declaration, 2002). The OSPAR Biodiversity Committee (BDC) was invited ‘to investigate the possibilities for further international cooperation in planning and managing marine activities through spatial planning of the North Sea States. The OPSAR Biodiversity Committee (BDC) adopted in 2007 the `Terms of Reference for an Intersessional Correspondence Group on Marine Spatial Planning` (ICG-MSP) with the aim of making a compilation of national spatial planning and control systems. The ecosystem approach has been reaffirmed in the Bergen Statement (2010) and MSP is considered as a tool to support this approach (OSPAR 10/23/1-E, Annex 49). In 2011 the ICG-MSP was re-established and its Term of Reference (ToR) adopted (OSPAR Commission, 2011, Annex 11; OSPAR Commission, 2011, Annex 17). The North East Atlantic Environmental Strategy 2010–2020` required the further development of appropriate measures, in line with the ecosystem approach, to facilitate MSP in the OSPAR maritime area (OSPAR 10/23/1-E, Annex 25). From 2012 onwards, any direct or indirect link to a potential OSPAR recommendations for a transboundary MSP disappeared from the agenda of the OSPAR COP meetings. The scope of the work of ICG-MSP 2014–2015 was to provide a platform for the exchange of best practices and experiences related to MSP. MSP remained the responsibility of governments and the focus of ICG-MSP should be on issues and working methods that are relevant in a transboundary context. The major role of IGG-MSP was facilitating the MSP learning process. | MSP: Workshops on Spatial Planning in the North Sea (SPINS)(2004). After the two SPINS workshops with focus on the North Sea, three workshops on Marine Spatial Management (MASMA) followed in 2005, 2006 and 2007 extending their scope outside the North Sea. MSP within OSPAR became a dead end and the EU took the MSP lead. An ICG-MSP was established in 2011. | Seabedwater columnsurface | Territorial sea, EEZ, continental shelfs | Exploitation, environmental conservation | Coastal states: main focus of spatial planning of the North Sea should be at the national level’ (OSPARCOM 2004, 04/5/1-E, para. 6). | - | - | see MSP North Sea by national authorities at https://www.msp-platform.eu/sea-basins/north-sea-0 and Ireland at https://www.msp-platform.eu/countries/ireland |
| 42 | MSP | In | UNESCO – IOC (note this is not at convention - entry into force is not relevant) | 2006 1st MSP Conference | Marine spatial planning (MSP) is a public process of analyzing and allocating the spatial and temporal distribution of human activities in marine areas to achieve ecological, economic, and social objectives that are usually specified through a political process. | MSP - The first MSP portal was developed after the First International Workshop on MSP organized by Fanny Douvere and Charles Ehler at UNESCO in November 2006. | Seabedwater columnsurface | Territorial sea, EEZ, High seas, continental shelfS | Exploration, exploitation, navigation, fisheries (aquaculture), environmental conservation and protection | Coastal states | MSP: charts, coordinates, maps, | For an overview of MSP global applications see http://msp.ioc-unesco.org/world-applications/overview/ | http://msp.ioc-unesco.org/about/msp-at-unesco/ |
| 43 | MSP | R | HELCOM (Baltic Sea): MSP | 2010 | 2010 HELCOM Ministerial Declaration on the implementation of the HELCOM Baltic Sea Action Plan: MSP should be developed for the different Baltic Sea areas in close transboundary cooperation with the aim of having long-term sustainable management and planning for the whole Baltic Sea. The Ministers agreed to establish a joint, co-chaired HELCOM-VASAB (Visions and Strategies around the Baltic Sea) WG on Maritime Spatial Planning enabling coordination and integration of MSP related actions and projects within the framework of the EU Strategy for the Baltic Sea Region and its Action Plan. This joint WG HELCOM-VASAB was asked to finalize a set of joint HELCOM-VASAB broad-scale transboundary MSP principles by 2010. HELCOM agreed to test, apply and evaluate the use of these joint principles when developing national MSP initiatives, as well as regional ones (HELCOM Ministerial Declaration, p. 4). | MSP | Seabed water columnsurface | Territorial sea, EEZ, continental shelfs | Exploitation, environmental conservation | Coastal state | MSP: GIS, maps | see http://maps.helcom.fi/website/mapservice/?datasetID=aa96bca9-23f5-4e24-bc92-be24cf101d59 or https://www.msp-platform.eu/sea-basins/baltic-sea-0 | https://vasab.org/ http://www.helcom.fi/action-areas/maritime-spatial-planning/msp-data/ |
| 44 | MSP | In | UNEP | 2014 - UNEP Report for CBD - See also CBD Decisions | Marine spatial planning  MSP) Marine spatial planning (MSP) is a participatory, multi-sectoral decision framework for allocating and regulating marine and coastal space to address the impacts of cumulative and potentially conflicting human activities to ensure a healthy environment and sustainable resource use into the future. | MSP: GEF-STAP and UNEP,2014, Marine spatial planning in practice, transitioning from planning to implementation. UNEP/CBD/SBSTTA/18/INF/23. 18 June 2014. | Any | Territorial sea, EEZ, continental shelf | Sustainable use of marine resources and to ensure a healthy environment and sustainable resource use into the future. | Sovereign states | MSP: coordinates, maps, plans | See GEF-STAP and UNEP[7] | 〈https://www.cbd.int/doc/meetings/sbstta/sbstta-18/information/sbstta-18- |
| 45 | MSP | R | European Union | 2014 | ‘Maritime spatial planning’ means a process by which the relevant Member State’s authorities analyze and organize human activities in marine areas to achieve ecological, economic and social objectives. | MSP: European Union Framework Directive on Maritime Spatial Planning 2014/89/EU, art. 3(2) | Seabedwater columnsurface | Territorial sea, EEZ, continental shelf | Exploitation non-living resources, navigation, aquaculture, environmental conservation and protection | MS | MSP: coordinates, maps, plans | For the state of implementation of the MSPD see https://www.msp-platform.eu/msp-eu/introduction-msp | https://ec.europa.eu/maritimeaffairs/policy/maritime_spatial_planning_en |
| 46 | U | In | UNESCO Convention concerning the Protection of World Cultural and Natural Heritage (1972); Recommendation concerning the Protection, at National Level, of the Cultural and Natural Heritage (1972) and the World Heritage Marine Programme | 1975 | Article 2. For the purposes of this Convention, the following shall be considered as `natural heritage': - natural features consisting of physical and biological formations or groups of such formations, which are of outstanding universal value from the aesthetic or scientific point of view; - geological and physiographical formations and precisely delineated areas which constitute the habitat of threatened species of animals and plants of outstanding universal value from the point of view of science or conservation; - natural sites or precisely delineated natural areas of outstanding universal value from the point of view of science, conservation or natural beauty. | Park, atoll, reef, lagoon, island, sanctuary, archipelago, reserve, ….: maps, charts, coordinates | Any | Inland waters, coastal waters, territorial sea, EEZ | In situ protection, natural beauty | The state(s) proposing a world heritage site and the World Heritage Committee decides to add the site to the list. Marine sites to be included in the world heritage list. World Heritage sites are selected through a rigorous, multi-year nomination, evaluation and inscription process. Outstanding Universal Value is the central premise upon which World Heritage is built. | No | There are 49 World Heritage Sites: Aldabra Atoll, Archipiélago de Revillagigedo, Area de Conservación Guanacaste, Banc d'Arguin National Park, Belize Barrier Reef Reserve System, Brazilian Atlantic Islands: Fernando de Noronha and Atol das Rocas Reserves, Cocos Island National Par, Coiba National Park and its Special Zone of Marine Protection, East Rennell, Everglades National Park Galápagos Islands, Gough and Inaccessible Islands Great Barrier Reef, Gulf of Porto: Calanche of Piana, Gulf of Girolata, Scandola Reserve, Ha Long Bay, Heard and McDonald Islands, High Coast / Kvarken Archipelago, Ibiza, Biodiversity and Culture, iSimangaliso Wetland Park, Islands and Protected Areas of the Gulf of California, Kluane / Wrangell-St. Elias / Glacier Bay / Tatshenshini-Alsek, Komodo National Park, Lagoons of New Caledonia: Reef Diversity and Associated Ecosystems, Lord Howe Island Group, Macquarie Island, Malpelo Fauna and Flora Sanctuary, Natural System of Wrangel Island Reserve, New Zealand Sub-Antarctic Islands, Ningaloo Coast Ogasawara Islands, Papahānaumokuākea, Península Valdés, Phoenix Islands Protected Area, Puerto-Princesa Subterranean River National Park Rock Islands Southern Lagoon, Sanganeb Marine National Park and Dungonab Bay, Mukkawar Island Marine National Park, Shark Bay, Western Australia, Shiretoko, Sian Ka'an, Socotra Archipelago, St Kilda, Sundarbans National Park Surtsey, The Sundarbans, Tubbataha Reefs Natural Park, Ujung Kulon National Park, Wadden Sea, West Norwegian Fjords – Geirangerfjord and Nærøyfjord, Whale Sanctuary of El Vizcaino | http://whc.unesco.org/en/marine-programme/ |
| 47 | U | In | UNESCO Convention on the Protection of Underwater Cultural Heritage (2001) | 2009 | States Parties should preserve underwater cultural heritage and take action accordingly. The in situ preservation of underwater cultural heritage (i.e. in its original location on the seafloor) should be considered as the first option before allowing or engaging in any further activities. The recovery of objects may, however, be authorized for the purpose of making a significant contribution to the protection or knowledge of underwater cultural heritage. The Convention neither regulates the ownership of wrecks nor does it change existing maritime zones. Wrecks and other cultural heritage sites underwater can be important for certain species as a small habitat. | Charts, coordinates | Seabed | Internal waters, contiguous zone, territorial sea, archipelagic water, continental shelf, EEZ, Area | In situ protection | Coastal states | No. Indirectly, if included in MSP (cf. Belgium) | The Meeting of Parties invited States Parties to the Convention to provide examples of Best Practices. The examples have hence to be submitted through the Permanent Delegation of a State that has ratified the 2001 Convention. Upon proposal of a Best Practice example it will be reviewed and evaluated by the Convention’s Scientific and Technical Advisory Body (STAB) with a view to designate the example as Best Practice, or not.  The following criteria apply:  a. the heritage falls under the definition of Article 1 of the 2001 Convention or is less than 100 years of age, but is classified as underwater cultural heritage according to national law,  b. the heritage is appropriately protected both legally and in practice; in particular, through the implementation of the Rules,  c. responsible non-intrusive access is respected,  d. the heritage has a framework to ensure sustainable management; and  e. a special and outstanding effort has been made to make the site accessible to the public.  (Source: <http://www.unesco.org/new/en/culture/themes/underwater-cultural-heritage/underwater-cultural-heritage/best-practices-of-underwater-cultural-heritage/>) | http://www.unesco.org/new/en/culture/themes/underwater-cultural-heritage/2001-convention/ |

**References**

[1] D.Y.C. Leung, G. Caramanna, M.M. Maroto-Valer, An overview of current status of carbon dioxide capture and storage technologies, Renew. Sustain. Energy Rev. 39 (2014) 426–443. https://doi.org/10.1016/j.rser.2014.07.093.

[2] B. Metz, B. Davidson, H. de Coninck, M. Loos Leo Meyer, Carbon dioxide capture and storage. Intergovernmental Panel on Climate Change, Cambridge, UK, 2005. https://www.ipcc.ch/site/assets/uploads/2018/03/srccs_wholereport-1.pdf (accessed March 25, 2020).

[3] S. Nanda, S.N. Reddy, S.K. Mitra, J.A. Kozinski, The progressive routes for carbon capture and sequestration, Energy Sci. Eng. 4 (2016) 99–122. https://doi.org/10.1002/ese3.117.

[4] Scientific Technical and Economic Committee for Fisheries (STECF), Monitoring the performance of the Common Fisheries Policy (STECF-Adhoc-18-01), Ispra, Italy, 2018. https://doi.org/10.2760/329345.

[5] European Commission, Report from the European Commission to the European Parliament and the Council on the progress in establishing marine protected areas, Bruxelles, Belgium, 2015. https://ec.europa.eu/environment/marine/eu-coast-and-marine-policy/implementation/pdf/marine_protected_areas.pdf.

[6] Comisiòn Permanente del Pacìfico Sur (CPPS), Red Regional de àreas costeras y marinas protegidas del Pacìfico Sudeste (Colombia, Chile, Ecuador, Panamà y Perù), Guayaquil, Ecuador, 2010. http://cpps-int.org/cpps-docs/pda/areas/docs/Red.regional.AMCP.PSE.2010.pdf (accessed March 27, 2020).

[7] GEF-STAP and UNEP, Marine Spatial Planning in Practice - Transitioning from Planning to Implementation, 2014.
